# Vertical stratification of bacteria and archaea in sediments of a small boreal humic lake

**DOI:** 10.1093/femsle/fnz044

**Published:** 2019-02-26

**Authors:** Antti J Rissanen, Sari Peura, Promise A Mpamah, Sami Taipale, Marja Tiirola, Christina Biasi, Anita Mäki, Hannu Nykänen

**Affiliations:** 1 Tampere University, Faculty of Engineering and Natural Sciences, Korkeakoulunkatu 10, FI-33720, Tampere, Finland; 2 University of Jyväskylä, Department of Biological and Environmental Science, PO Box 35, FI-40014, Jyväskylä, Finland; 3 University of Eastern Finland, Department of Environmental and Biological Sciences, PO Box 1627, FI-70211, Kuopio, Finland

**Keywords:** lake, sediment, bacteria, archaea, 16S rRNA, biomass

## Abstract

Although sediments of small boreal humic lakes are important carbon stores and greenhouse gas sources, the composition and structuring mechanisms of their microbial communities have remained understudied. We analyzed the vertical profiles of microbial biomass indicators (PLFAs, DNA and RNA) and the bacterial and archaeal community composition (sequencing of 16S rRNA gene amplicons and qPCR of *mcrA*) in sediment cores collected from a typical small boreal lake. While microbial biomass decreased with sediment depth, viable microbes (RNA and PLFA) were present all through the profiles. The vertical stratification patterns of the bacterial and archaeal communities resembled those in marine sediments with well-characterized groups (e.g. *Methanomicrobia*, *Proteobacteria*, *Cyanobacteria*, *Bacteroidetes*) dominating in the surface sediment and being replaced by poorly-known groups (e.g. *Bathyarchaeota*, *Aminicenantes* and *Caldiserica*) in the deeper layers. The results also suggested that, similar to marine systems, the deep bacterial and archaeal communities were predominantly assembled by selective survival of taxa able to persist in the low energy conditions. Methanotrophs were rare, further corroborating the role of these methanogen-rich sediments as important methane emitters. Based on their taxonomy, the deep-dwelling groups were putatively organo-heterotrophic, organo-autotrophic and/or acetogenic and thus may contribute to changes in the lake sediment carbon storage.

## Teaser

DNA sequencing and biomass analysis show strong vertical stratification of microbial communities in sediments of a small humic boreal lake.

## INTRODUCTION

A large number of small humic forest lakes are typical for the boreal region (Downing *et al*. 2006). They are globally significant carbon stores and hot spots of processes producing greenhouse gases, especially methane (Molot and Dillon 1996; Bastviken *et al*. 2004; Kortelainen *et al*. 2004; Kankaala *et al*. 2007; Wik *et al*. 2016). As microbes are important mediators of biogeochemical processes, data on the composition and structuring mechanisms of the bacterial and archaeal communities in these lakes is crucial in the study of global carbon cycling and in mitigation and prediction of climate change.

Lake sediment microbial communities are generally understudied relative to their marine counterparts (Petro *et al*. 2017). The studies have also mostly focused on lakes in the temperate area, and information on sediment microbial communities of small boreal lakes is scarce (Koizumi, Kojima and Fukui 2003; Ye *et al*. 2009; Borrel *et al*. 2012; Wurzbacher *et al*. 2017). Sediments of marine systems and lakes are characterized by increasing energy limitation, i.e. increasing contribution of recalcitrant organic matter and decreasing availability of electron acceptors, with depth, which can also cause variation in their microbial communities (Petro *et al*. 2017; Wurzbacher *et al*. 2017). However, the vertical stratification patterns of the biomass (abundance) and composition of microbial communities in the sediments of small boreal lakes was not explored in previous studies (Steger *et al*. 2011; Youngblut, Dell'Aringa and Whitaker 2014; Rissanen *et al*. 2017). In marine sediments, the abundance of microbes decrease downwards alongside with increasing energy limitation (Petro *et al*. 2017; Starnawski *et al*. 2017). Marine sediments are also characterized by a steep vertical stratification in the microbial community composition with well-characterized taxonomic groups (groups for which functional data and many cultivated representatives exist) being replaced by poorly-known groups (groups for which negligible/scant functional data and no or only a few cultivated representatives exist) when moving deeper in the sediment (Petro *et al*. 2017; Starnawski *et al*. 2017). Instead, the few existing lake sediment studies from the temperate area indicate that the stratification patterns of the abundance and composition of microbial communities differ between lakes (Koizumi, Kojima and Fukui 2003; Ye *et al*. 2009; Borrel *et al*. 2012; Wurzbacher *et al*. 2017). Similar to marine sediments, the composition of bacterial and archaeal communities changed considerably and the abundance of microbes (concentration of DNA and cells) decreased with sediment depth in Lake Stechlin (Wurzbacher *et al*. 2017). In contrast, despite drastic vertical change in the composition of archaea, the abundance of bacteria and archaea (16S rRNA gene copy number) did not change with depth in Lake Pavin (Borrel *et al*. 2012), and the abundance of bacteria even increased but their composition did not change with depth in Lake Taihu (Ye *et al*. 2009). However, the vertical stratification patterns of the sediment communities of small humic-rich boreal lakes cannot be predicted based on these results, as they differ considerably from the temperate lakes in the physicochemical conditions that structure the microbial communities, for example in the quality and quantity of organic matter (Xiong *et al*. 2015). For instance, variations in dissolved organic carbon (DOC) concentrations indicate that both the quantity of organic matter and the contribution of non-labile allochtonous organic matter is larger in small humic-rich boreal lakes (up to 25 mg C L^−1^) than in the previously studied temperate lakes (up to 1.8–7 mg C L^−1^) (Mothes, Koschel and Proft 1985; Viollier *et al*. 1995; Sugiyama *et al*. 2005; Ye *et al*. 2015). Because methane produced in the sediments accumulate in high amounts in the lower parts of the water column (Houser *et al*. 2003; Kankaala *et al*. 2007; Peura *et al*. 2012), it can be expected that methanogens are especially abundant and methanotrophs rare in the sediments of small boreal lakes. However, further studies are needed to confirm this as well as to explore, whether and how the bacterial and archaeal community changes in the low-energy conditions below the zone dominated by methanogens.

The mechanisms controlling the vertical variations in microbial community composition in sediments of lakes are also unclear. Evidence from marine sediments suggests that the transition from the surface to deep subsurface biosphere is due to filtering (i.e. selection) of populations from the surface that leave only a subset of taxa to populate the deeper energy limited zones (Petro *et al*. 2017). This mechanism was shown by a low number of operational taxonomic units (OTUs) that are present at all depths (i.e. persisting OTUs) but make up a significant proportion of the total sediment communities (Petro *et al*. 2017). It may well explain the vertical distribution of bacterial and archaeal communities also in lakes, but has not been previously investigated.

We wanted to fill the knowledge gap on sediment microbial communities of small humic-rich boreal lakes. Therefore, we characterized the vertical variation in bacterial and archaeal communities in sediments (26 cm deep cores) of a small boreal humic forest lake (Lake Alinen Mustajärvi) via DNA/RNA quantification, next-generation sequencing of 16S rRNA gene amplicons, phospholipid fatty acid (PLFA)—analysis and quantitative-PCR (qPCR) of methyl coenzyme M reductase (*mcrA*) gene (a biomarker gene of methanogenic archaea). Furthermore, the vertical variation in the sediment organic matter quality was assessed via analyzing the content and stable isotopic ratios of carbon and nitrogen of the bulk sediment. Our main aim was to resolve the vertical change in the bacterial and archaeal community composition and in the microbial biomass alongside with the increasing energy limitation (i.e. increasing organic matter recalcitrance) with depth in the study lake sediments. We also specifically aimed to verify our expectation that methanogens are abundant and methanotrophs rare in the sediments of the study lake. Furthermore, we aimed to elucidate whether the vertical change in bacterial and archaeal communities in the sediments of the study lake are predominantly controlled by the same mechanism as in their marine counterparts, i.e. by selective survival of taxa able to persist under the energy limitation.

## MATERIALS AND METHODS

### Study lake

Lake Alinen Mustajärvi (A = 0.7 ha, max. depth = 6.5 m, V = 31 000 m^3^ and DOC = ∼10–20 mg C L^−1^) is a small, humic, headwater lake located in southern Finland (61°12’N; 25°06’E). The catchment area is <0.5 km^2^ and consists of >90% coniferous forest and <10% peatland. The lake is ice covered each year from late November to late April. Alinen Mustajärvi is spring meromictic. Thus, the water column mixes completely only during autumn. The lake water column is stratified with respect to temperature and oxygen during both summer and winter. Most parts of the year, the sediment surface is exposed to anoxic and cold hypolimnion water with relatively stable temperature (4–6°C) (Nykänen *et al*. 2014).

### Sampling

Sampling was done through holes drilled in ice at the deepest point of the lake on Apr 4th 2012. Water column profiles of temperature and oxygen concentrations were measured *in situ* using a portable field meter (YSI model 58, Yellow Springs Instruments, Yellow Springs, Ohio, USA). The sediment surface was exposed to anoxic and cold water (4.5°C) during the time of sampling representing typical conditions of the sediments for most of the year. Sediment samples were collected using a slicing (height of the slicing ring = 1 cm) Limnos Sediment Sampler (Limnos.pl, Komorów, Poland) (length = 94 cm, Ø = 9.4 cm) connected to a gravity corer. Altogether, two cores (thus, n = 2, length 26 cm from the water-sediment interface), were collected from 3 meters apart from each other. For the 0–20 cm zone (0 cm = water-sediment interface), the cores were divided into 1 cm thick layer-specific samples (i.e. layers: 0–1 cm, 1–2 cm, …18–19 cm, 19–20 cm), except that layer 15–16 cm was not sampled. For the 20–26 cm zone, the cores were divided into 2 cm thick samples (i.e. 20–22 cm, 22–24 cm and 24–26 cm). Immediately after collection, the samples were homogenized and subsamples were collected from each sample for DNA/RNA extraction and for determination of dry weight as well as content and isotopic composition of C and N of bulk sediment. The sediments were stored frozen (at outdoor temperature, −19°C) until transported to the laboratory. Samples were merged into pools corresponding to 2 to 6 cm thick depth layers (20 samples, 10 layers per core) for phospholipid fatty acid (PLFA) analysis (layers: 0–2 cm, 2–4 cm, 4–6 cm, 6–8 cm, 8–10 cm, 10–12 cm, 12–15 cm, 16–18 cm, 18–20 cm and 20–26 cm). All the samples were then stored frozen (at –20°C) before further analyses which took place within 1–2 months from sampling. Sediment age of the study lake is not known. However, sediment age has been determined for a closely located (within 5 km distance in the same forest area) Lake Valkeakotinen, which represents a similar type of small, shallow (max. depth 6.7 m), humic, spring-meromictic headwater lake. Based on these analyses, a 26 cm long sediment core represent approximately 200 years of sediment accumulation (Wickstrom and Tolonen 1987; Pajunen 2004).

### Analysis of chemical sediment qualities

Subsamples of frozen sediment were freeze-dried for determination of the dry weight of each layer. Freeze-dried sediment was also homogenized and weighted into small tin cups for analysis of content and stable isotope ratios of C and N using a Thermo Finnigan Flash EA1112 elemental analyser connected to a Thermo Finnigan DELTAplus Advantage continuous-flow stable isotope ratio mass spectrometer (Thermo Fisher Scientific, Waltham, Massachusetts, USA). Isotopic composition of C and N is expressed in terms of δ value, which is parts per thousand differences from a standard (Vienna Pee Dee Belemnite for C and atmospheric N_2_ for N): δ^13^C or δ^15^N = [(R_sample_/R_standard_)-1]*10^3^, where R is the ^13^C/^12^C or ^15^N/^14^ N—ratio.

### PLFA extraction and analysis

Total lipids of the freeze-dried samples were extracted and fractionated into neutral, glyco-, and phospholipids, and the phospholipid fraction was analyzed as explained before (Bligh and Dyer 1959; Mpamah *et al*. 2017). The amount (µg fatty acids g^−1^ dw) of BrFA (sum of i14:0, i15:0, a15:0, i16:0, i17:0, a17:0 and i18:0) was used as a biomarker of living bacterial biomass. BrFA were chosen for this purpose as they are specifically of bacterial origin, generally related to gram-positive prokaryotes, sulphate-reducing bacteria and other anaerobic bacteria in sediments (Zhukova 2005).

### Nucleic acid—based analyses

DNA and RNA were simultaneously extracted from frozen sediment samples (190–340 mg) using a previously published protocol based on bead-beating and phenol–chloroform extraction (Griffiths *et al*. 2000). Nucleic acid yields of extractions were determined with Qubit 2.0 Fluorometer and Qubit™ dsDNA HS Assay Kit for DNA and Qubit™ RNA HS Assay Kit for RNA (Thermo Fisher Scientific). DNA extractions were stored at −20°C before sequencing and qPCR analyses.

The abundance of methanogenic archaea was studied via qPCR of the *mcrA* gene as described in detail in Supplemental Methods (Supplemental Methods, Supporting Information). Furthermore, the community structure of bacteria and archaea was studied by next-generation sequencing of bacterial and archaeal 16S rRNA gene amplicons. Primers, PCR, preparation of NGS libraries, and the sequencing (Ion Torrent™ Personal Genome Machine) are described in detail in Supplemental Methods. Sequencing analysis of archaeal 16S rRNA genes was done from 43 samples representing almost all the collected depth-layers from the two sample cores. However, for sequencing analysis of bacterial 16S rRNA genes and for qPCR analysis, equal amount of DNA from each sample were pooled into samples corresponding to the same depth layers as in PLFA analysis (20 samples, 10 layers per core, see above).

### Bioinformatic analyses

Mothur was used in subsequent sequence analyses (Schloss *et al*. 2009). Barcodes and primer sequences, as well as low-quality sequences (containing sequencing errors in primer or barcode sequences, ambiguous nucleotides and homopolymers longer than eight nucleotides) were removed. Thereafter, alignment, chimera-removal, pre-clustering (to reduce PCR-amplification and sequencing errors), taxonomic classification (Silva v128 database), division of sequences into OTUs (at 97% similarity level), singleton removal and subsampling of each sample to the size of the smallest sample were conducted as previously described (Rissanen *et al*. 2018). Sequences classified as chloroplast, mitochondria and eukaryota were removed from all libraries. Furthermore, bacterial sequences were removed from archaeal libraries and vice versa. Finally, there were 1239 and 11 837 archaeal and bacterial sequences per sample with an average length of 270 and 308 bp, respectively. Sequence variation was adequately covered in these libraries as shown by Good's coverage that varied 0.91–0.97 and 0.88–0.95 for archaeal and bacterial libraries, respectively.

Bacterial and archaeal OTUs were classified into 1) well-characterized groups (groups with functional data and cultivated representatives) and 2) poorly-known groups (groups with negligible/scant functional data and either no or only a few cultured representatives) according to Table 1. We also specifically studied the contribution of methanogens and methanotrophs in the archaeal and bacterial communities. Furthermore, to assess whether the deep bacterial and archaeal communities predominantly assemble by selective survival of different taxa, we analyzed the number of surface layer OTUs surviving depth-wise from layer to layer, and the relative abundance of persisting OTUs (i.e. OTUs present in each layer) (Petro *et al*. 2017; Starnawski *et al*. 2017). For the analyses, the values of the replicate samples were averaged for each layer. Furthermore, archaea were studied with similar layering as bacteria by averaging the values of sample layers of archaeal samples (e.g. 0–1 cm and 1–2 cm layers) to represent layering of bacterial samples (e.g. 0–2 cm layer). For comparison, we conducted a similar analysis of assembly mechanisms of bacterial and archaeal communities for sediments of Lake Stechlin via reanalysis of a recently published dataset (Additional File 11 in Wurzbacher *et al*. 2017).

**Table 1.** Well-characterized and poorly-known taxa detected in the study lake sediments using 16S rRNA gene sequencing.

Group	*Archaea*	*Bacteria*
**Well-characterized**		
	Classes:	Phyla:
	*Methanobacteria*, *Methanomicrobia*	*Acidobacteria*, *Actinobacteria*, *Armatimonadetes*, *Bacteroidetes*, *Chlamydiae*, *Cyanobacteria*, *Deinococcus-Thermus*, *Fibrobacteres*, *Firmicutes*, *Fusobacteria*, *Gemmatimonadetes*, *Lentisphaerae*, *Nitrospirae*, *Planctomycetes*, *Proteobacteria*, *Spirochaetae*, *Verrucomicrobia*
**Poorly-known** ^a^		
	unclassified *Archaea*	unclassified *Bacteria*
	Classes:	Phyla:
	*Thermoplasmata*	*AC1*, *Acetothermia*, *Aminicenantes*, *Atribacteria*
	Phyla:	*Berkelbacteria*, *BRC1*
	*Aenigmarchaeota*, *Altiarchaeales*, *Bathyarchaeota*, *Diapherotrites*, *Hadesarchaea*, *Lokiarchaeota*, *Miscellaneous Euryarchaeotic Group (MEG)*, *Parvarchaeota*, *Thaumarchaeota*, *Woesearchaeota, YNPFFA*	*Caldiserica*, *Chlorobi*, *Chloroflexi*, *Cloacimonetes*, *Elusimicrobia*, *FCPU426*, *Gracilibacteria*, *Hydrogenedentes*, *Ignavibacteriae*, *Latescibacteria*, *LCP-89*, *Microgenomates*, *Omnitrophica*, *Parcubacteria*, *Peregrinibacteria*, *RBG-1*, *Saccharibacteria*, *SR1*, *TA06*, *TM6*, *WS1*, *WS2*, *WS6*

a The detected 16S rRNA gene sequences assigned to *Chlorobi* were not from the known phototrophic genera nor were *Chloroflexi*—sequences from known organohalide respiring genera or *Thermoplasmata*—sequences from known methanogenic genera. This supports the classification of these taxa into the group of poorly-known microbes.

### Accession numbers

16S rRNA gene sequences were deposited in NCBI´s short read archive under accession number SRP120305.

## RESULTS AND DISCUSSION

There were vertical variations in the bulk sediment C%, N%, C:N—ratio, δ^15^N and δ^13^C, which can be caused by variations in organic matter input and in diagenetic processes (Fig. S1, Supporting Information). We lack information on the history of the lake to assess the relative importance of these factors. However, the generally higher C:N and δ^15^N and lower δ^13^C in deep than in surface layers can be expected to reflect decomposition and, thus, increased energy limitation due to increased organic matter recalcitrance with depth. This is caused by preferential microbial degradation of labile, high-nitrogen organic compounds with high δ^13^C leaving the residual organic matter ^15^N-enriched and ^13^C-depleted (Fig. S1, Supporting Information) (Lehmann *et al*. 2002). Of recalcitrant organic compounds, especially lignin, which is an important component of organic matter leaching to freshwater systems in the boreal area, could lead to low δ^13^C in sediments (Benner *et al*. 1987; Amon *et al*. 2012).

**Figure 1.**
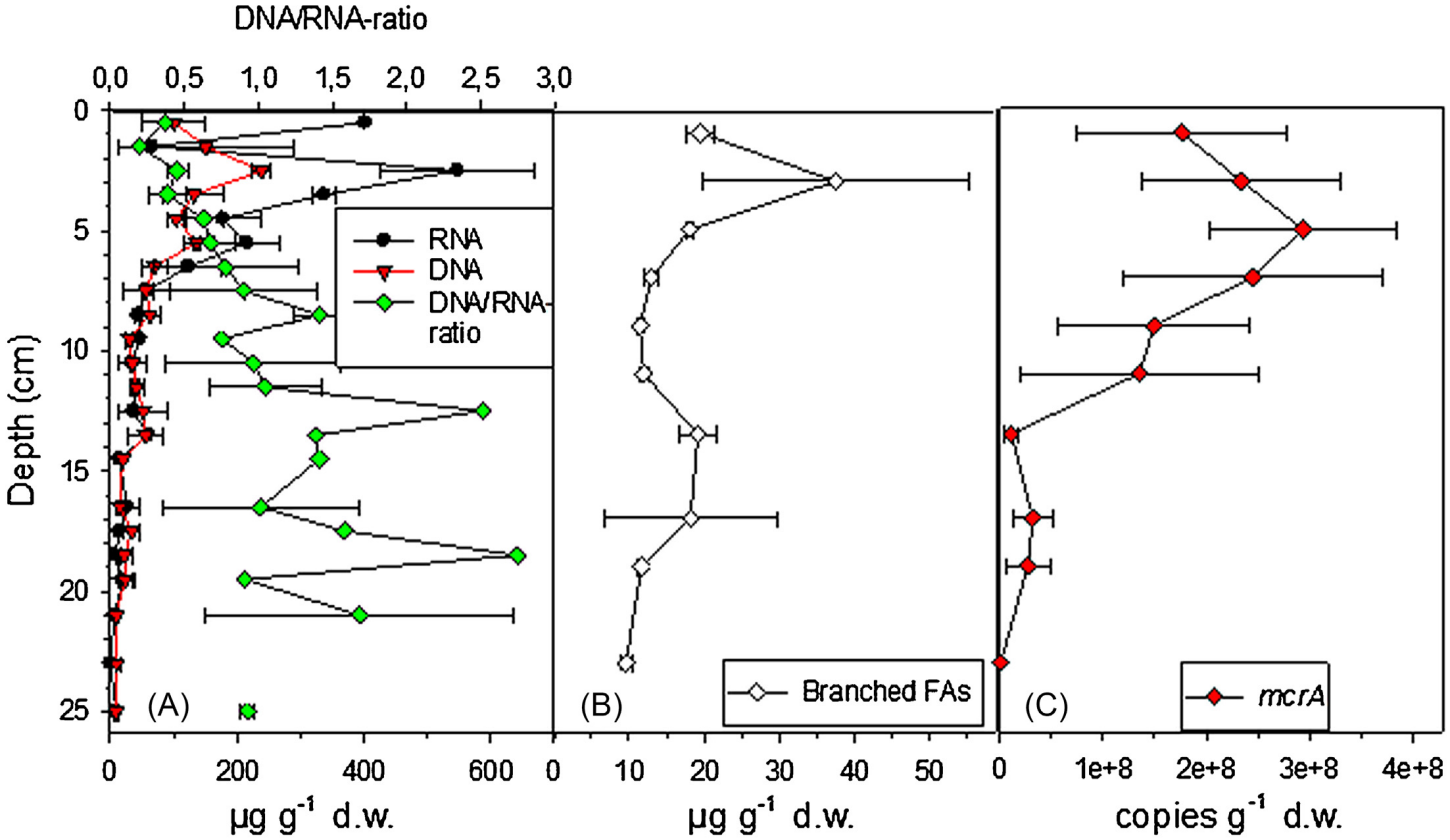
Vertical variation in the **(A)** amount of RNA and DNA as well as in the DNA:RNA—ratio, **(B)** amount of BrFA (sum of i14:0, i15:0, a15:0, i16:0, i17:0, a17:0 and i18:0) in PLFA fraction and **(C)***mcrA* gene copy numbers in the sediments of the study lake. Results represent mean of two replicate cores and their average deviation. Depth of each data point is the average depth of the particular study layer. DNA:RNA—ratio could not be calculated for the layer 22–24 cm because RNA was below detection limit (in A).

Based on the content of DNA, RNA and branched fatty acids (BrFA) of the sediment, the microbial biomass and abundance was highest at the top 4 cm below which it generally decreased until the deepest layer of the sample cores (Fig. 1A and B). Decrease in biomass and abundance was caused by downward increasing energy limitation agreeing with results from marine and some lake systems (Haglund *et al*. 2003; Petro *et al*. 2017; Starnawski *et al*. 2017; Wurzbacher *et al*. 2017). However, the presence of biomarkers of viable microbes (i.e. RNA and fatty acids, Blagodatskaya and Kuzyakov 2013) down to the deepest layers of the sediment core indicate that the conditions in the deep layers supported microbial life (Fig. 1A and B). This agrees with Haglund *et al*. (2003) showing active cells down to 25 cm depth in sediments of Lake Erken. Interestingly, the depth-wise decrease was more profound with nucleic acids than with BrFAs (Fig. 1A and B). Similarly, DNA content decreased much more than cell numbers with sediment depth in Lake Stechlin (Wurzbacher *et al*. 2017). As nucleic acids originate from both eukaryotes and prokaryotes and BrFAs only from bacteria, the difference in the vertical distribution of nucleic acids and BrFAs may be partially explained by steeper decrease in eukaryotic biomass than in bacterial biomass (Wurzbacher *et al*. 2017). However, the generally larger vertical reduction in DNA than in cell numbers (Lake Stechlin) and bacterial BrFAs (our study) can be also due to vertical reduction in cell-size and cell-specific DNA contents. Decrease in cell and genome size could be a consequence of adaptation to nutrient limited conditions in the deep layers (Giovannoni, Thrash and Temperton 2014). In fact, as described below, the relative abundance of taxa featuring small genome size increased in the deeper sediment.

**Figure 2.**
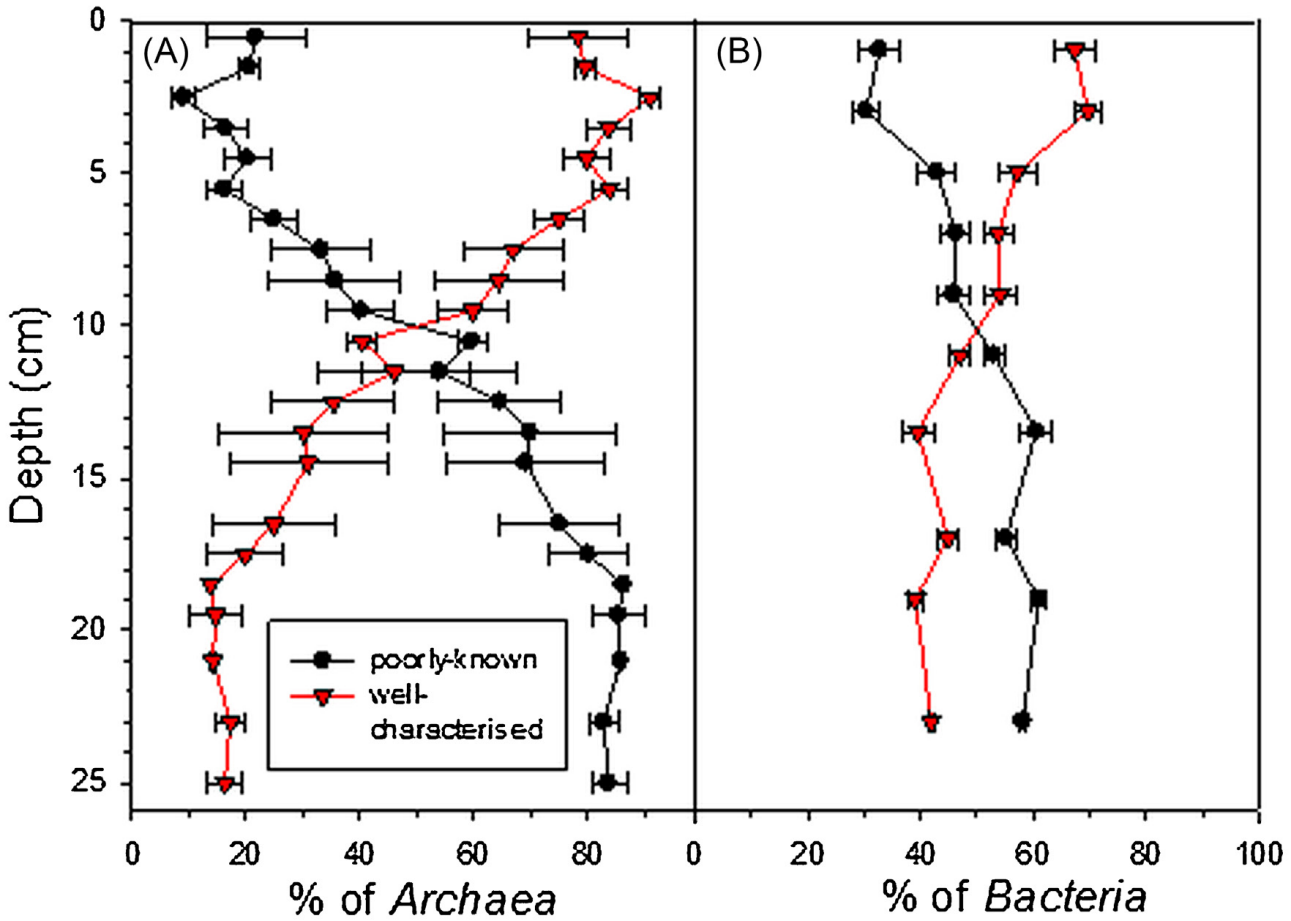
Vertical variation in the sum of the relative abundances of poorly-known and well-characterized **(A)** archaeal and **(B)** bacterial taxa, including also unclassified *Archaea* (in A) and *Bacteria* (in B), in the sediments of the study lake based on 16S rRNA gene sequencing. Results represent mean of two replicate cores and their average deviation. Depth of each data point is the average depth of the particular study layer. See Table 1 for the list of taxa in the study lake sediments.

**Figure 3.**
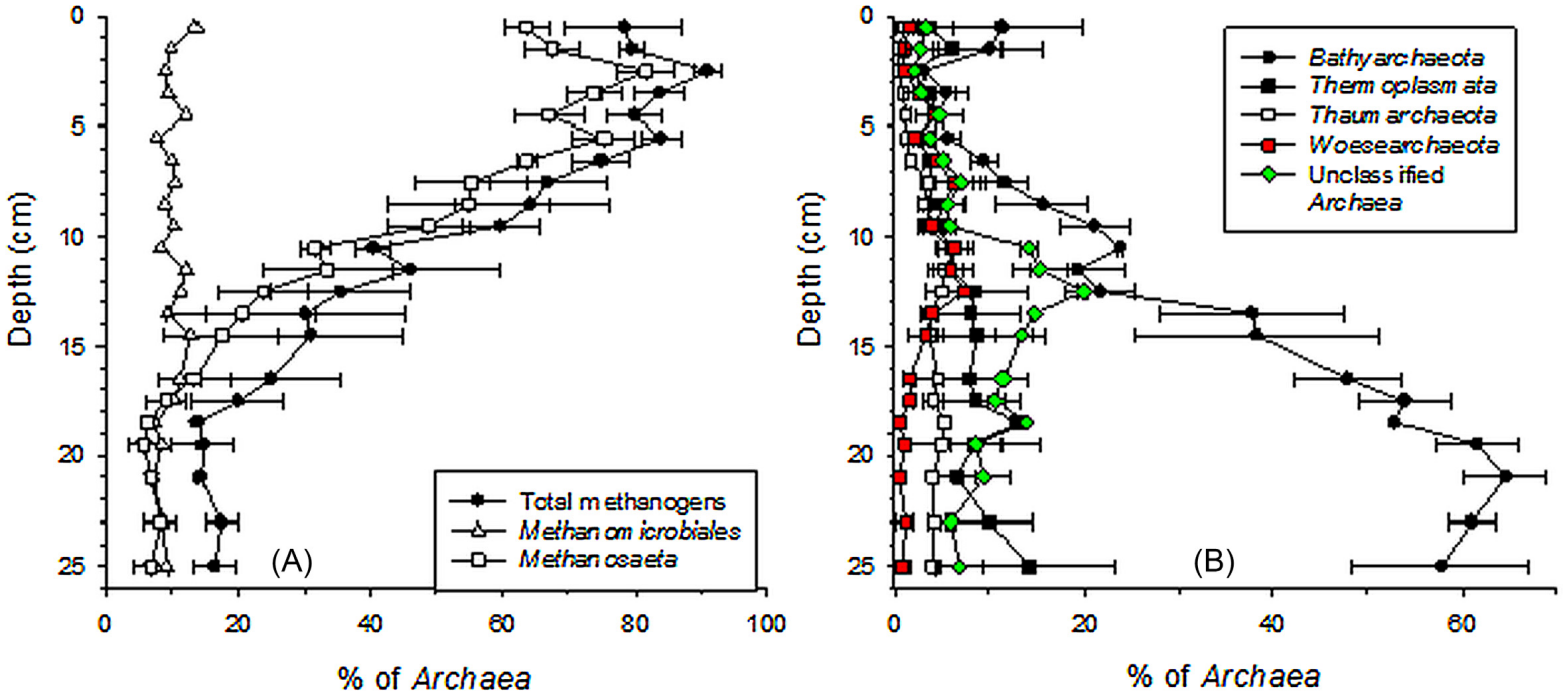
Vertical variation in the relative abundance of dominant (i.e. consisting > 1% of the archaeal 16S rRNA gene sequences in the dataset) **(A)** well-characterized archaeal (i.e. euryarcheotal methanogens), and **(B)** poorly-known archaeal taxa as well as unclassified *Archaea* in the sediments of the study lake based on 16S rRNA gene sequencing. Results represent mean of two replicate cores and their average deviation. Depth of each data point is the average depth of the particular study layer.

**Figure 4.**
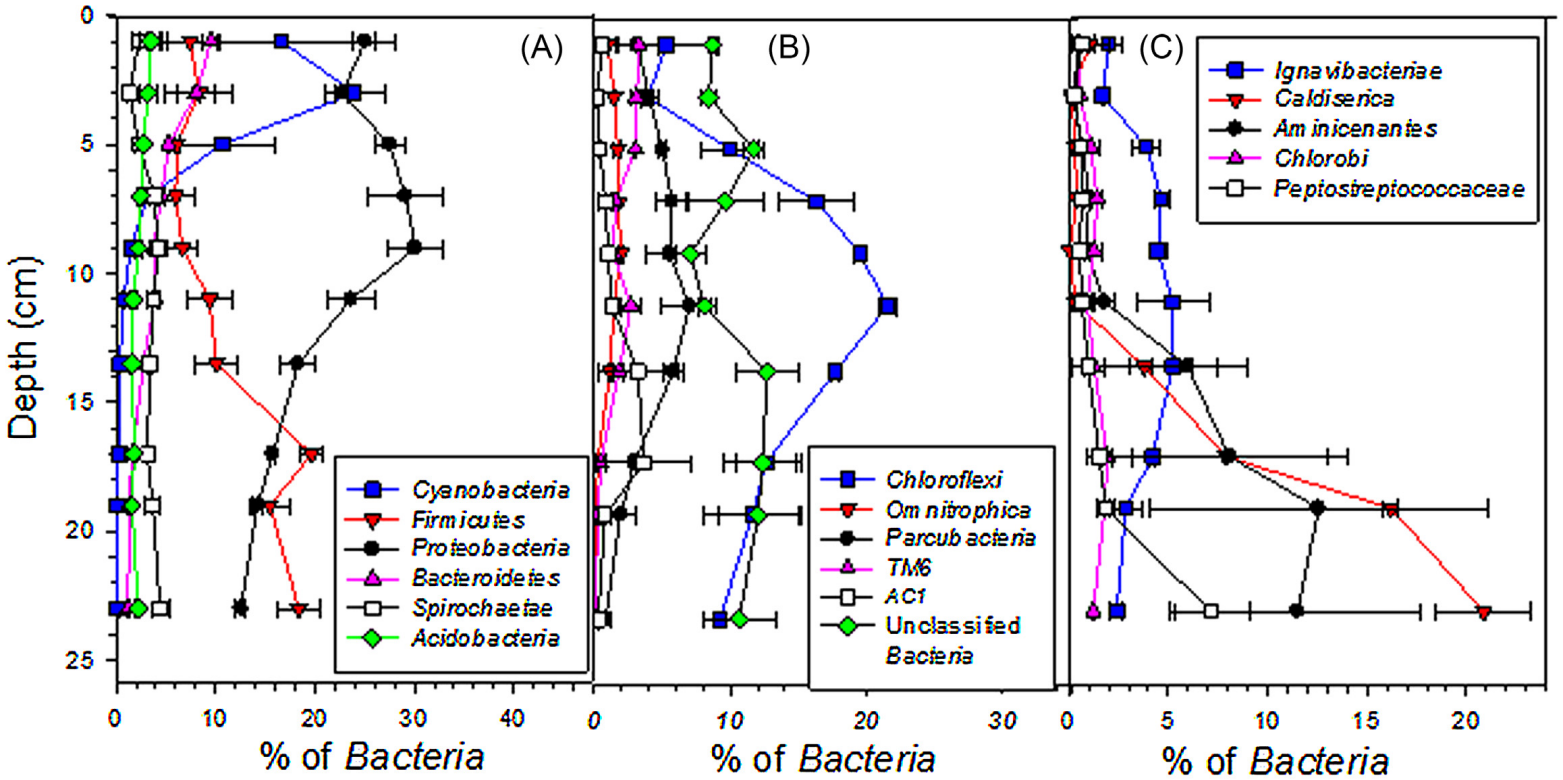
Vertical variation in the relative abundance of **(A)** dominant (i.e. consisting > 1% of the bacterial 16S rRNA gene sequences in the dataset) well-characterized bacterial phyla, **(B)** and **(C)** dominant poorly-known bacterial phyla and unclassified *Bacteria*, as well as, **(C)** a putative protein degrading family *Peptostreptococcaceae* (*Firmicutes*), in the sediments of the study lake based on 16S rRNA gene sequencing. Results represent mean of two replicate cores and their average deviation. Depth of each data point is the average depth of the particular study layer.

Sediment bacterial and archaeal communities were vertically stratified with some poorly-known taxonomic groups increasing deeper in the sediment (Figs 2-4), which agrees with results from temperate lakes (Borrel *et al*. 2012; Wurzbacher *et al*. 2017) and from marine systems (Petro *et al*. 2017; Starnawski *et al*. 2017). This suggests that it is a widespread pattern in many types of organic sediment ecosystems. However, the possible effect of relic DNA on the diversity estimates has to be acknowledged (Carini *et al*. 2016). Indeed, the depth-wise increase in DNA:RNA—ratio could reflect increasing amount of relic DNA (Fig. 1A). However, increase in DNA:RNA—ratio can be also due to microbes being less active and producing less RNA in deep than in surface layers. Similar to Wurzbacher *et al*. (2017), we suggest that extractable relic DNA in our study lake was very short-lived. Furthermore, relic DNA is expected to mostly affect the results on the rare biosphere (Wurzbacher *et al*. 2017), which we did not target in our study.

As expected, methanogens belonging to the phyla *Euryarchaeota* dominated archaeal communities in the upper sediment layers (Fig. 3A). The vertical variation in *mcrA* abundance further confirmed that methanogens were very important in the top 14 cm layer (Fig. 1C). The largest poorly-known archaeal group was *Bathyarchaeota*, which dominated the lower layers (Fig. 3B). Other dominant poorly-known archaeal groups (i.e. consisting > 1% of the archaeal 16S rRNA gene sequences in the dataset) were unclassified *Archaea*, *Thaumarchaeota* and *Thermoplasmata*, which also had generally higher relative abundance in the deep than in the surface layers, as well as *Woesearchaeota*, which had its highest relative abundance in the middle of the sediment core (Fig. 3B). The dominant groups of well-characterized bacteria (i.e. consisting > 1% of bacterial 16S rRNA gene sequences in the dataset) consisted of *Proteobacteria*, *Cyanobacteria*, *Bacteroidetes* and *Acidobacteria* which had generally higher relative abundance in the surface layers than in the deep layers, as well as of *Firmicutes* and *Spirochaetae*, which showed contrasting depth patterns (Fig. 4A). Of the dominant groups of poorly-known bacteria, *Caldiserica*, *Aminicenantes*, *Chlorobi* and unclassified *Bacteria* had a higher relative abundance in the deep layers than in the surface layers, while other groups either decreased towards bottom, i.e. *TM6* and *Omnitrophica*, or showed an elliptical distribution pattern, i.e. *Chloroflexi, Parcubacteria*, *AC1* and *Ignavibacteriae* (Fig. 4B and C). Comparison of the results with previous literature also suggest that genome sizes of the bacterial and archaeal cells decreased with sediment depth. Published genome sizes of the largest deep sediment phyla, *Caldiserica*, 1.6 Mb (*Caldicericum exile*, KEGG genome database), *Aminicenantes*, 2.5 Mb (metagenome assembled genome in Robbins *et al*. 2016) and *Bathyarchaeota*, ∼0.6–1.9 Mb (Castelle *et al*. 2018) are within the lower end of range reported for the largest surface layer phyla, *Proteobacteria*, ∼0.2–10 Mb, *Cyanobacteria*, ∼1.6–9 Mb, *Bacteroidetes*, ∼0.3–6 Mb and *Euryarchaeota*, ∼1.6–5.9 Mb (Castelle *et al*. 2018).

Putative acetoclastic (acetate—consuming) methanogens (*Methanosaeta*) dominated over hydrogenotrophic (H_2_ and CO_2_–consuming) methanogens (*Methanomicrobiales*) with a much higher ratio (up to 9:1) than the theoretically predicted 2:1 for methane production in acetoclastic:hydrogenotrophic pathways in complete methanogenic degradation of organic matter (Fig. 3A) (Conrad 1999). This could be due to low temperature (4–6°C for most of the year) and pH (5–6) in the lake bottom increasing the relative contribution of acetoclastic pathway via increase in the bacterial acetate production (Phelps and Zeikus 1984; Glissmann *et al*. 2004; Nykänen *et al*. 2014). *Methanosaeta*—methanogens may have also partially sustained their metabolism through CO_2_–reduction linked to direct interspecies electron transfer (Rotaru *et al*. 2014). The downward changes in the relative contribution of acetoclastic and hydrogenotrophic methanogens was a further indication of increasing organic matter recalcitrance in deeper depths (Fig. 3A) (Conrad, Claus and Casper 2009). Furthermore, half of *Proteobacteria* consisted of fermentative *Deltaproteobacteria* (*Syntrophobacterales* and *Syntrophorhabdaceae*). In addition, fermentative function has been proposed for *Firmicutes*, *Bacteroidetes*, *Parcubacteria*, *TM6*, *Chloroflexi* and *Ignavibacteriae* (McInerney *et al*. 2008; Iino *et al*. 2010; Wrighton et al., 2012, 2014; Hug *et al*. 2013; Kantor *et al*. 2013; Podosokorskaya *et al*. 2013; Kallistova, Goel and Nozhevnikova 2014; Wasmund *et al*. 2014). Thus, a significant proportion of sediment bacteria potentially provided substrates for methanogenesis.

As expected, methanotrophs did not make a significant contribution to the sediment bacterial and archaeal community (Fig. S2). In fact, anaerobic methane oxidizing bacteria [i.e. *NC10*-phyla (Ettwig *et al*. 2010)] or archaea [i.e. ANME—archaea (Knittel and Boetius 2009)] or aerobic verrucomicrobial methanotrophs were not detected in the sediments. Archaeal genus *Methanosarcina*, whose member, *M. acetivorans*, can potentially mediate anaerobic methane oxidation, was also very rare, only up to 0.25% of archaeal 16S rRNA genes (Fig. S2B, Supporting Information) (Yan *et al*. 2018). In addition, aerobic methanotrophs in the order *Methylococcales* and in the family *Methylocystaceae* consisted only up to 0.6% and 1.7% of bacterial 16S rRNA gene sequences, respectively (Fig. S2A, Supporting Information). These results differ from those of the water column of the same study lake, where *Methylococcales* were up to 16% of bacteria (Rissanen *et al*. 2018). They also disagree with those from sediments of larger boreal lakes, where aerobic methanotrophs had a higher relative abundance (i.e. *Methylococcales* were up to 8% and *Methylocystaceae* up to 4% of bacteria) and which harbored also anaerobic methane oxidizing bacteria and archaea (Rissanen *et al*. 2017). However, *Methylococcales* in the water column of the study lake resided close to the oxic-anoxic interface (Rissanen *et al*. 2018). Furthermore, the sediments of the larger dimictic boreal lakes usually have an oxic surface all the year around, whereas the water layers above the sediment surface of small spring-meromictic boreal lakes are mostly completely anoxic, except during autumn-mixing. Thus, the lower and temporally more variable availability of oxygen explained the low relative abundance of aerobic methanotrophs in the sediments of the study lake. Furthermore, profiles of sulfate and hydrogen sulfide indicate active sulfate reduction in the anoxic water columns of small boreal humic lakes suggesting that the electron acceptors used in anaerobic methane oxidation are exhausted for most of the year (Schiff *et al*. 2017; Rissanen *et al*. 2018). This very likely prevented the establishment of populations of anaerobic methane oxidizing bacteria and archaea in the sediments of the study lake. Anaerobic methanotrophs were also absent in the water column of the study lake (Rissanen *et al*. 2018). In contrast, aerobic methanotrophs sustained their life also during the long anoxic periods, yet the type of their anaerobic metabolism is mostly uncharacterized (Roslev and King 1994; Bar-Or *et al*. 2017; Martinez-Cruz *et al*. 2017).

**Figure 5.**
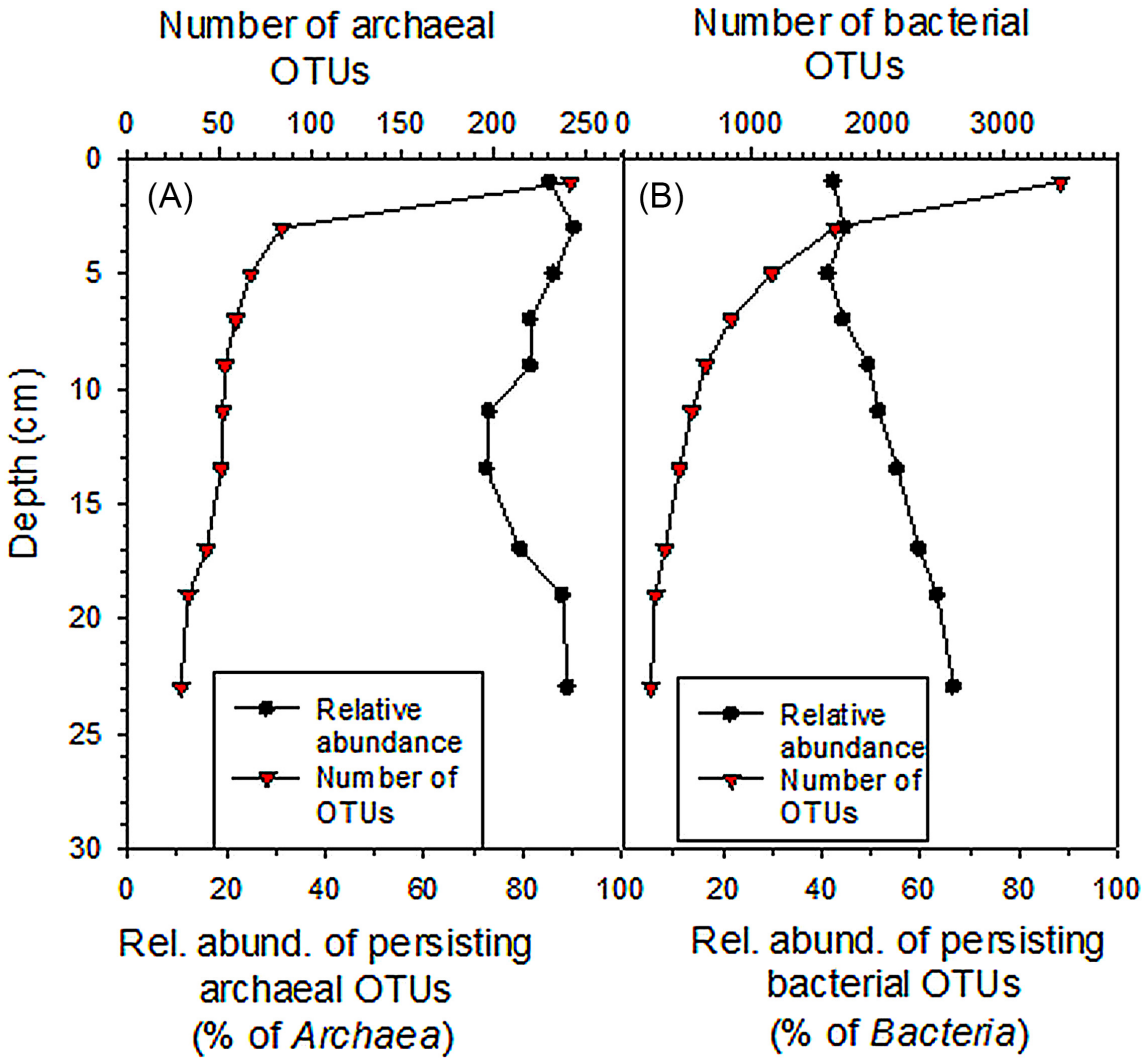
Vertical distribution in the number of surface layer OTUs surviving depth-wise from layer to layer, and in the relative abundance of persisting OTUs (i.e. OTUs present in each layer from top to bottom) in the sediments of the study lake for **(A)***Archaea* and **(B)***Bacteria*, based on 16S rRNA gene sequencing. Thus, number of OTUs at the top-most layer represent number of all the OTUs at the surface, whereas number of OTUs at the bottom-most layer represent number of persisting OTUs (i.e. OTUs present in each layer from top to bottom). Depth of each data point is the average depth of the particular study layer.

The OTUs, that were present in all depth layers (persisting OTUs), had only very minor contribution to the total richness (5%–9% and 5%–17% of the number of bacterial and archaeal OTUs), yet constituted a large fraction of the communities (41%–67% and 73%–90% of bacterial and archaeal sequences, respectively) (Fig. 5). This agrees with results from marine sediments (Petro *et al*. 2017; Starnawski *et al*. 2017). The persisting OTUs represented the same taxonomic groups as the dominant groups of poorly-known and well-characterized bacteria and archaea (e.g. *Cyanobacteria*, *Caldiserica*, *Methanosaeta* and *Bathyarchaeota*), and, thus, played a significant role in structuring the major vertical patterns in the microbial community (Figs S3, Supporting Information; Figs 3 and 4). In accordance, our reanalysis of the dataset from Wurzbacher *et al*. (2017) showed that, in Lake Stechlin, the persisting OTUs had only minor contribution to the total richness (2%–9% and 4%–11% of bacterial and archaeal OTUs) but still constituted a significant fraction of the communities (29%–41% and 35%–71% of bacterial and archaeal sequences) (Fig. S4A and B, Supporting Information). Altogether, this suggests that the bacterial and archaeal communities in the deep sediments of the lakes are predominantly assembled by the same mechanism as in their marine counterparts, i.e. by selective survival of taxa able to persist under the energy limitation. The community assembly mechanism could not be inferred for other previously studied lake sediments because they were done using low-resolution methods (DGGE or TTGE) (Koizumi, Kojima and Fukui 2003; Ye *et al*. 2009; Borrel *et al*. 2012).

The dominant deep sediment phyla, *Caldiserica*, *Aminicenantes* and *Bathyarcheota* are typical members of freshwater systems, including lakes (Borrel *et al*. 2012; Faraq *et al*. 2014; Wurzbacher *et al*. 2017). The relative abundance of these phyla also increased with depth in Lake Stechlin (Fig. S4C, Supporting Information) (Wurzbacher *et al*. 2017), and an archaea-specific study showed a similar pattern for *Bathyarcheota* in Lake Pavin (Borrel *et al*. 2012). Furthermore, *Bathyarchaeota* is a key microbial phylum in deep marine sediments (Starnawski *et al*. 2017). Thus, members of these phyla are adapted to living in low-energy conditions. Blastn-searches confirmed that the *Bathyarchaeota* in the study lake were not closely related to the putative methanogenic members of this phylum (Altschul *et al*. 1990; Evans *et al*. 2015). Instead, previous metagenomic, single-cell genomic and culture-dependent analyses suggest that members of *Bathyarchaeota*, *Caldiserica* and *Aminicenantes* are organo-heterotrophs, yet, organo-autotrophic and acetogenic lifestyle has been also proposed for some members of *Bathyarchaeota* (Mori *et al*. 2009; Lloyd *et al*. 2013; Rinke *et al*. 2013; He *et al*. 2016; Lazar *et al*. 2016; Yu *et al*. 2018). As recently shown by Yu *et al*. (2018) for marine sediment *Bathyarchaeota* and suggested by our stable isotopic data (see above, Fig. S1A, Supporting Information), lignin might be an important energy source for the *Bathyarchaeota* in the sediments of the study lake and generally in sediments of lakes having forested catchments. However, protein-degrading function has been also specifically proposed for both *Bathyarchaeota* and *Aminicenantes* (Mori *et al*. 2009; Lloyd *et al*. 2013; Rinke *et al*. 2013; Lazar *et al*. 2016). In support of this, a well-characterized protein-mineralizing family, *Peptostreptococcaceae* (Slobodkin 2014), showed a concurrent downward increase in its relative abundance in the study lake (Fig. 4C). Yet, the same did not take place in Lake Stechlin (Wurzbacher *et al*. 2017) (Fig. S4C, Supporting Information). It is still tempting to speculate that the capability for protein degradation, for example via degrading dead microbial biomass or stable organometallic complexes that contain proteins and other labile organic matter (Lalonde *et al*. 2012), would be an advantageous trait in low energy conditions. Furthermore, besides energy metabolism based on fermentation and acetogenesis, as suggested for *Bathyarchaeota* (He *et al*. 2016; Lazar *et al*. 2016), anaerobic respiration linked to reduction of poorly reactive Fe(III) minerals may also become a thermodynamically favorable process in electron acceptor—poor conditions below the methanogenesis zone in lake sediments (Bar-Or *et al*. 2017). Consequently, sulfur-respiration could be also promoted in the deep sediments via abiotic sulfide oxidation linked to iron reduction. In support of that, the only cultured representative of *Caldiserica*, *Caldisericum exile*, do not ferment but grows by anaerobic respiration reducing thiosulfate, sulfite and elemental sulfur (Mori *et al*. 2009).

## CONCLUSION

Based on results from the boreal study lake and the previously studied temperate lakes, it can be concluded that lake sediment bacterial and archaeal communities generally follow a similar stratification pattern as communities in marine systems with the relative importance of poorly-known groups increasing with depth. The results also suggest that, similar to marine systems, the bacterial and archaeal communities in deep sediment layers of lakes are predominantly assembled by selective survival of taxa able to persist in the low energy conditions. The dominance of fermentative bacteria and methanogenic archaea and the rarity of methanotrophs in the study lake adds to the growing body of evidence on the role of the sediments of small boreal humic lakes as important methane emitters. As the poorly-known, deep-dwelling groups potentially contribute to changes in the lake sediment carbon store, future studies utilizing culture-independent and –dependent study methods are needed to resolve their activities in lake sediments and under different physicochemical conditions.

## Supplementary Material

Supplemental Files
