# Supplementary material for: Vertical stratification of bacteria and archaea in sediments of a small boreal humic lake
Source: FEMS Microbiol Lett. 2019 Feb 26;366(5):fnz044. doi: 10.1093/femsle/fnz044 (PMC6476745; doi:10.1093/femsle/fnz044)
Supplement: Supplemental Files [file fnz044_supplemental_files.zip › Rissanen_Supporting_information_FigS1-S4.docx]

**Supporting Information, Figs. S1 – S4**

**Fig. S1**

**Figure S1**. Vertical variation in **(A)** Stable isotopic ratios of carbon (δ^13^C) and nitrogen (δ^15^N), **(B)** C and N content (in %), and **(C)** C/N – ratio of the bulk sediment in the study lake. Results represent mean of two replicate cores and their average deviation. Depth of each data point is the average depth of the particular study layer.

**Fig. S2**

**Figure S2.** Vertical variation in the relative abundance of **(A)** aerobic methanotrophic bacteria, i.e. order *Methylococcales* and family *Methylocystaceae*, and **(B)** putative anaerobic methanotrophic archaea, i.e. *Methanosarcina*, in the sediments of the study lake based on 16S rRNA gene sequencing. Results represent mean of two replicate cores and their average deviation. Depth of each data point is the average depth of the particular study layer.

**Fig. S3**

**Figure S3.** Vertical variation in the relative abundance of persisting OTUs (i.e. OTUs present in each layer), summed for each dominant taxa, in the sediments of the study lake for **(A)** *Archaea* and **(B)** *Bacteria*, based on 16S rRNA gene sequencing. Depth of each data point is the average depth of the particular study layer.

**Fig. S4**

**Figure S4.** Vertical distribution in the number of surface layer OTUs surviving depth-wise from layer to layer, and in the relative abundance of persisting OTUs (i.e. OTUs present in each layer from top to bottom) for **A)** *Archaea* and **B)** *Bacteria*, as well as, **C)** vertical distribution in the relative abundance (average +/- SD, n = 4) of *Bathyarcheaota*, *Caldiserica*, *Aminicenantes* and *Peptostreptococcaceae*, in the sediments of Lake Stechlin based on 16S rRNA gene sequencing. Thus, number of OTUs at the top-most layer represent number of all the OTUs at the surface, whereas number of OTUs at the bottom-most layer represent number of persisting OTUs (i.e. OTUs present in each layer from top to bottom) (in A & B). Depth of each data point is the average depth of the particular study layer. The figure is based on re-analysis of data presented as Additional File 11 in Wurzbacher *et al*. (2017).
