# Supplementary material for: Vertical stratification of bacteria and archaea in sediments of a small boreal humic lake
Source: FEMS Microbiol Lett. 2019 Feb 26;366(5):fnz044. doi: 10.1093/femsle/fnz044 (PMC6476745; doi:10.1093/femsle/fnz044)
Supplement: Supplemental Files [file fnz044_supplemental_files.zip › Rissanen_Supporting_information_Supplemental_Methods.docx]

**Supporting Information, Supplemental Methods**

**Quantitative PCR**

The abundance of methanogenic archaea was studied by qPCR of the *mcrA* gene using primer pair Mlas-F (5’-GGTGGTGTMGGDTTCACMCARTA-3’) / mcrA-rev (5´-CGTTCATBGCGTAGTTVGGRTAGT-3´) (Steinberg and Regan 2008, 2009). Amplification of qPCR and fluorescent data collection was carried out with a Bio-Rad CFX96 thermal cycler (Bio-Rad, Hercules, California, USA) in a reaction mixture of 0.5 μM of both primers, 12.5 μl Maxima SYBR Green/Fluorescein qPCR Master Mix (Thermo Fisher Scientific), 1 µl DNA extract, and PCR-grade water to yield a total volume of 25 μl. Two replicate qPCR amplifications were performed for each sample. The PCR procedure included an initial denaturation step at 95°C for 10 min and 40 cycles of amplification (95°C for 30 s, 55°C for 45 s and 72°C for 30 s). Finally, an increase of 0.05°C/s from 50 to 95°C was performed to obtain the melting curve analysis of PCR products. Standard curves were constructed from PCR amplicons extracted from agarose gel with a Gel Extraction Kit (Bio-Rad). Amplicons were re-amplified and the resulting products were purified with Agencourt AMPure XP (Beckman Coulter, Indianapolis, Indiana, USA). A dilution series of 10^7^–10^2^ gene copies were used as standards in each qPCR run. Inhibition was tested from the dilution series (1, 1:10 and 1:100) and no inhibition was detected.

**Sequencing**

PCR of 16S rRNA gene amplicons was done using domain-specific, universal primer pairs Arch340F (5’- CCCTAYGGGGYGCASCAG – 3’) / Arch1000R (5’- GGCCATGCACYWCYTCTC – 3’) for archaea (Gantner *et al.* 2011) and 27F (5’ – AGAGTTTGATCMTGGCTCAG - 3’) / 338R (5’ – TGCTGCCTCCCGTAGGAGT – 3’) for bacteria. For archaeal and bacterial 16S rRNA, two PCR reactions were done. In the first reaction for archaea, ~3 ng of DNA was used as a template in a 26 µl PCR mixture containing 0.2 mM of dNTPs, 0.3 µM of both primers, 1x Phusion Buffer, 0.4 mg ml^-1^ BSA and 0.03 U µl^-1^ Phusion High-Fidelity DNA polymerase (Thermo Fisher Scientific). In the first reaction for bacteria, ~9 ng of DNA was used as a template in a 40 µl PCR mixture containing 0.25 mM of dNTPs, 0.25 µM of both primers, 1x Phusion Buffer and 0.03 U µl^-1^ Phusion High-Fidelity DNA polymerase. PCR amplification of both archaeal and bacterial 16S rRNA gene was performed in C1000™ Thermal Cycler (Bio-Rad) with an initial denaturation step at 98ºC for 30 sec and 30 cycles of amplification (98ºC for 10 sec, 53ºC for 30 sec, 72ºC for 60 sec). In the second PCR, 1 µl (archaea) or 0.5 µl (bacteria) of PCR product from the first PCR, were used as a template in 26 µl (archaea) or 20 µl (bacteria) PCR mixture. PCR conditions were similar as above except that all primers but Arch1000R included sequencing adaptors (A adaptor linked with 27F and Arch340F; P1 adaptor linked with 338R) as well as nucleotide barcodes incorporated between the A adapter and forward primers to distinguish samples in the mixed reaction and only 8 (archaea) or 6 (bacteria) PCR-amplification cycles were run. PCR products were purified with Agencourt AMPure XP, measured for their DNA concentration using Qubit 2.0 Fluorometer and Qubit™ dsDNA HS Assay Kit for DNA (Thermo Fisher Scientific), and pooled in equal DNA amounts for both genes. Amplicon pool was further purified using Agencourt AMPure XP. Fragmentation of the archaeal 16S rRNA gene amplicons into length suitable for sequencing, library preparation as well as sequencing using Ion Torrent PGM (Thermo Fisher Scientific) was done as previously explained (Mäki, Rissanen and Tiirola 2016; Rissanen *et al.* 2017).

**References**

Gantner S, Andersson AF, Alonso-Sáez L *et al.* Novel primers for 16S rRNA-based archaeal community analyses in environmental samples. *J Microbiol Methods* 2011;**84**:12–8.

Mäki A, Rissanen AJ, Tiirola M. A practical method for barcoding and size-trimming PCR templates for amplicon sequencing. *Biotechniques* 2016;**60**:88–90.

Rissanen AJ, Karvinen A, Nykänen H *et al.* Effects of alternative electron acceptors on the activity and community structure of methane-producing and consuming microbes in the sediments of two shallow boreal lakes. *FEMS Microbiol Ecol* 2017;**93**:fix078.

Steinberg LM, Regan JM. Phylogenetic comparison of the methanogenic communities from an acidic, oligotrophic fen and an anaerobic digester treating municipal wastewater sludge. *Appl Environ Microbiol* 2008;**74**:6663–71.

Steinberg LM, Regan JM. *mcrA*-targeted real-time quantitative PCR method to examine methanogen communities. *Appl Environ Microbiol* 2009;**75**:4435–42.
